# Inducibility of the endogenous antibiotic peptide β-defensin 2 is impaired in patients with severe sepsis

**DOI:** 10.1186/cc5694

**Published:** 2007-02-15

**Authors:** Malte Book, QiXing Chen, Lutz E Lehmann, Sven Klaschik, Stefan Weber, Jens-Christian Schewe, Markus Luepertz, Andreas Hoeft, Frank Stuber

**Affiliations:** 1 Department of Anaesthesiology and Intensive Care Medicine, Rheinische-Friedrich-Wilhelms University Bonn, Sigmund-Freud-Str. 25, 53105 Bonn, Germany; 2 Department of Anaesthesiology, School of Medicine, Zhejiang University, 388 Yuhang Tang Road, 310058 Hangzhou, People's Republic of China

## Abstract

**Introduction:**

The potent endogenous antimicrobial peptide human β-defensin 2 (hBD2) is a crucial mediator of innate immunity. In addition to direct antimicrobial properties, different effects on immune cells have been described. In contrast to the well-documented epithelial β-defensin actions in local infections, little is known about the leukocyte-released hBD2 in systemic infectious disorders. This study investigated the basic expression levels and the *ex vivo *inducibility of hBD2 mRNA in peripheral whole blood cells from patients with severe sepsis in comparison to non-septic critically ill patients and healthy individuals.

**Methods:**

This investigation was a prospective case-control study performed at a surgical intensive care unit at a university hospital. A total of 34 individuals were tested: 16 patients with severe sepsis, 9 critically ill but non-septic patients, and 9 healthy individuals. Serial blood samples were drawn from septic patients, and singular samples were obtained from critically ill non-septic patients and healthy controls. hBD2 mRNA levels in peripheral white blood cells were quantified by real-time polymerase chain reaction in native peripheral blood cells and following *ex vivo *endotoxin stimulation. Defensin plasma levels were quantified by enzyme-linked immunosorbent assay.

**Results:**

Endotoxin-inducible hBD2 mRNA expression was significantly decreased in patients with severe sepsis compared to healthy controls and non-septic critically ill patients (0.02 versus 0.95 versus 0.52, *p *< 0.05, arbitrary units). hBD2 plasma levels in septic patients were significantly higher compared to healthy controls and critically ill non-septic patients (541 versus 339 versus 295 pg/ml, *p *< 0.05).

**Conclusion:**

In contrast to healthy individuals and critically ill non-septic patients, *ex vivo *inducibility of hBD2 in peripheral blood cells from septic patients is reduced. Impaired hBD2 inducibility may contribute to the complex immunological dysfunction in patients with severe sepsis.

## Introduction

Endogenous antimicrobial peptides are widely distributed in various species [1,2]. They are part of the innate immune system and their genes are highly conserved throughout the animal and plant kingdoms. In humans, antimicrobial defensins are divided into α- and β-defensins according to their molecular structure. They display a broad antimicrobial effect against bacteria, fungi, mycobacteria, and coated viruses [2-5]. Defensins act by permeabilising microbial membranes. In addition, β-defensins are chemotactic for immature dendritic cells and memory T cells. They regulate cytokine production and adhesion-molecule expression, stimulate epithelial cell and fibroblast proliferation, and promote histamine release from mast cells [6,7].

To date, six human β-defensin genes have been characterised and located on chromosome 8. The epithelial human β-defensin 1 (*hBD1*) gene is constitutively expressed at low levels and slightly upregulated following stimulation [8]. In contrast, *hBD2*, *hBD3*, and *hBD4 *gene expression is inducible mainly by various inflammatory stimuli in different cell types [9-12] The recently described *hBD5 *and *hBD6 *represent epididymis-specific human defensins [13].

There is increasing evidence for the clinical relevance of defensins. Alpha- and β-defensins contribute to anti-HIV activity [14,15]. In newborns, respiratory tract β-defensin mRNA expression is upregulated in response to infection [16]. Moreover, a systemic release of β-defensins in infectious diseases has been reported [17]. Our own previous experiments detected hBD2 mRNA expression in white blood cells following *ex vivo *stimulation by endotoxin [18]. In particular, systemic infection underlying syndromes such as severe sepsis challenges the immune system by constant activation of its adaptive and innate components. The responsiveness of the innate immune system, including expression of endogenous antibiotic peptides like β-defensins, contributes to the final resolution of the disease.

The present study investigated hBD2 mRNA levels in native peripheral white blood cells as well as the *ex vivo hBD2 *mRNA inducibility in patients with severe sepsis. Additionally, we determined hBD2 protein plasma levels in patients. The hypothesis that hBD2 expression is disturbed in patients with severe sepsis was tested.

## Materials and methods

### Patients and controls

This study was performed according to the ethical standards stated in the 1964 Declaration of Helsinki. After approval by the local ethics committee and receipt of the written informed consent of either the patient or a close relative, 16 patients treated on a surgical intensive care unit (ICU) at a university hospital with the diagnosis of severe sepsis were included in this prospective case-control study. The diagnosis of severe sepsis met the criteria of the American College of Chest Physician/Society of Critical Care Medicine Consensus Conference Committee [19]. Exclusion criteria were (a) lack of informed consent, (b) age younger than 18 years, and (c) pre-existing immunological or haematological diseases. Whole blood samples were drawn on the day of diagnosis (day 1) and on the third and fifth days of severe sepsis. A fourth blood sample was drawn after recovery from severe sepsis at ICU discharge in survivors or at imminent death in the case of non-survivors (day X).

In addition, two control groups were included: nine non-septic critically ill ICU patients who were in need of intensive care and who were without any signs of infection (blood samples were drawn once during the ICU treatment) and nine healthy volunteers (blood samples were drawn once). All patients and volunteers were of German Caucasian origin.

### Blood culture and RNA isolation

Whole blood was co-cultured for four hours with 500 pg/ml lipopolysaccharide contained in the Milenia^® ^*ex vivo *stimulation kit (Milenia Biotec, Hohe Str. 4–8, 61231 Bad Nauheim, Germany) at 37°C and 5% CO_2_. After incubation, the blood was centrifuged at 1,500 *g *for five minutes. The supernatant was stored at -70°C for further analysis. Total RNA was extracted from whole blood by means of a QIAamp^® ^RNA Blood Kit (Qiagen, Hilden, Germany) according to the manufacturer's instructions and then dissolved in diethylpyrocarbonate-treated water and stored at -70°C until further analysis.

Basic *hBD2 *mRNA levels were investigated using Paxgene^® ^Blood RNA System tubes (PreAnalytiX; Qiagen GmbH, Hilden, Germany). For this analysis, 2.5 ml of whole blood was drawn in Paxgene^® ^tubes and treated as indicated in the manufacturer's instructions. By this method, intracellular RNA was stabilised until further analysis. RNA isolation was performed using the Paxgene^® ^kit according to the manufacturer's instructions.

### cDNA preparation

cDNA was produced as polymerase chain reaction (PCR) template using 1st Strand cDNA Synthesis Kit for RT-PCR^® ^(avian myeloblastosis virus [AMV]) (Roche Diagnostics, Sandhofer Str. 116, 68305, Mannheim, Germany). The reaction mixture contained 8.2 μl (approximately 500 ng) of total RNA, 5 mM MgCl_2_, 1 mM dNTP, 3.2 μg of random primer p(dN)_6_, 50 units of RNase inhibitor, 20 units of AMV reverse transcriptase, and 1× reaction buffer in a total volume of 20 μl. The reaction was incubated at 25°C for 10 minutes, 42°C for 60 minutes, and 99°C for 5 minutes and then cooled to 4°C for 5 minutes.

### Real-time PCR

The PCR was performed on a LightCycler^® ^instrument (Roche Diagnostics). For the amplification of *hBD2*, the reaction mixture included 10 μl of cDNA, 1 μM each primer (forward and reverse), 0.15 μM each hybridisation probe (labelled with fluorescein and LC-Red640; TIB MOLBIOL GmbH, Berlin, Germany), and 1× Lightcycler FastStart Master^PLUS ^Mix (Roche Diagnostics) in a total volume of 20 μl. For detection of the housekeeping gene *hHPRT *(human hypoxanthine phosphoribosyl-transferase), the 20 μl of reaction mixture consisted of 2 μl of cDNA, 2 μl of reaction mix for *hHPRT *(Roche), and 12 μl of ddH_2_O in 1× Lightcycler FastStart Master^PLUS ^Mix (Roche Diagonostics). The sequences of primers and hybridisation probes specific for *hBD2 *measurement were as follows: forward primer: 5'-CTGATGCCTCTTCCAGGTGT-3'; reverse primer: 5'-GGAGCCCTTTCTGAATCCG-3'; probes: 5'-GGTATAAACAAATTGGCACCTGTGGTC-FL and 5'-LC Red640 CCCTGGAACAAAATGCTGCAAAA-PH.

The PCRs for *hBD2 *and *hHPRT *were conducted in separate capillaries as duplicates. The reaction was performed as follows: initial denaturation at 95°C for 10 minutes followed by 45 cycles of 95°C for 5 seconds, 55°C for 8 seconds, and 72°C for 10 seconds. The reaction was then cooled at 40°C for 30 seconds. Fluorescence was monitored at the end of each 55°C incubation and detected in channel F2/F1. The crossing point (C_P_) of each reaction was analysed by the method of second derivative maximum algorithm (C_P _was defined as cycle number at detection threshold).

### Relative quantification analysis

The expression level of *hBD2 *mRNA in each sample was analysed by LightCycler Relative Quantification Software (Roche Diagnostics). The principles and workflows have been described previously [20]. In summary, the quantity of a target (*hBD2*) and a reference (*hHPRT*) gene is a function of the PCR efficiency and the sample C_P _and does not require a standard curve in each LightCycler analysis run for its determination. C_P _value is most reliably proportional to the initial template concentration. Differences in PCR efficiency result from different primers as well as hybridisation probes and can be corrected by the software. Results are expressed as the target/reference ratio of each sample normalised by the target/reference ratio of the calibrator. The calibrator is included in every run and its ratio is set to a value of 1. This normalisation provides a constant calibrator point between PCR runs.

Normalised ratio=conc.target (sample)con.reference (sample):conc.target (calibrator)conc.reference (calibrator),
 MathType@MTEF@5@5@+=feaafiart1ev1aaatCvAUfKttLearuWrP9MDH5MBPbIqV92AaeXatLxBI9gBaebbnrfifHhDYfgasaacH8akY=wiFfYdH8Gipec8Eeeu0xXdbba9frFj0=OqFfea0dXdd9vqai=hGuQ8kuc9pgc9s8qqaq=dirpe0xb9q8qiLsFr0=vr0=vr0dc8meaabaqaciaacaGaaeqabaqabeGadaaakeaacqqGobGtcqqGVbWBcqqGYbGCcqqGTbqBcqqGHbqycqqGSbaBcqqGPbqAcqqGZbWCcqqGLbqzcqqGKbazcqqGGaaicqqGYbGCcqqGHbqycqqG0baDcqqGPbqAcqqGVbWBcqGH9aqpdaWcaaqaaiabbogaJjabb+gaVjabb6gaUjabbogaJjabb6caUiabbsha0jabbggaHjabbkhaYjabbEgaNjabbwgaLjabbsha0jabbccaGiabcIcaOiabbohaZjabbggaHjabb2gaTjabbchaWjabbYgaSjabbwgaLjabcMcaPaqaaiabbogaJjabb+gaVjabb6gaUjabb6caUiabbkhaYjabbwgaLjabbAgaMjabbwgaLjabbkhaYjabbwgaLjabb6gaUjabbogaJjabbwgaLjabbccaGiabcIcaOiabbohaZjabbggaHjabb2gaTjabbchaWjabbYgaSjabbwgaLjabcMcaPaaacqGG6aGodaWcaaqaaiabbogaJjabb+gaVjabb6gaUjabbogaJjabb6caUiabbsha0jabbggaHjabbkhaYjabbEgaNjabbwgaLjabbsha0jabbccaGiabcIcaOiabbogaJjabbggaHjabbYgaSjabbMgaPjabbkgaIjabbkhaYjabbggaHjabbsha0jabb+gaVjabbkhaYjabcMcaPaqaaiabbogaJjabb+gaVjabb6gaUjabbogaJjabb6caUiabbkhaYjabbwgaLjabbAgaMjabbwgaLjabbkhaYjabbwgaLjabb6gaUjabbogaJjabbwgaLjabbccaGiabcIcaOiabbogaJjabbggaHjabbYgaSjabbMgaPjabbkgaIjabbkhaYjabbggaHjabbsha0jabb+gaVjabbkhaYjabcMcaPaaacqGGSaalaaa@BA81@

Normalised ratio = E_T_^CpT(C) - CpT(S) ^× E_R_^CpR(S) - CpR(C)^,

where E = efficiency of PCR amplification, T = target gene, R = reference gene, S = unknown sample, and C = calibrator.

In this experiment, the coefficient file was created by PCR amplification of *hBD2 *and *hHPRT *as the housekeeping gene in a series of diluted cDNA (relative standard curve) in triplicates. Data of real-time PCR, including calibrator and samples, were imported into the Relative Quantification Software and analysed with the Fit Coefficients File. Finally, the normalised ratios were calculated. These ratios directly reflect the expression level of hBD2 mRNA.

### hBD2 plasma protein quantification

Twenty micrograms of hBD2 polypeptides was diluted in acetic acid to form the 1 μg/μl stock solution and then adjusted to 10 mM Tris/0.5% bovine serum albumin (BSA)/0.05% Tween-20 to obtain serial concentrations of the *hBD2 *standard: 2,000 pg/ml, 1,000 pg/ml, 500 pg/ml, 250 pg/ml, 125 pg/ml, and 62.5 pg/ml. Samples were diluted in 1:4 dilution buffer 10 mM Tris/0.5% BSA/0.05% Tween-20. Coating of the standards and samples was performed in a 96-well plate with 100 μl of phosphate-buffered saline coating buffer at 4°C overnight.

Thereafter, the plates were blocked with 300 μl of 5% non-fat bovine milk blocking buffer at 37°C for 2 hours. The goat polyclonal β-defensin 2 antibody (Abcam plc, 332 Cambridge Science Park, Milton Road, Cambridge, UK) was diluted to 0.5 μg/ml with 5% non-fat bovine milk antibody dilution buffer. One hundred microlitres was applied to each well. After additional washing, the peroxidase-conjugated rabbit anti-goat immunoglobulin G antibody (1:1,200) (Sigma-Aldrich Chemie GmbH, Eschenstrasse 5, 82024 Taufkirchen, Germany) was applied to the wells. Plates were covered and incubated at 37°C for two hours. Washing was followed by the addition of 100 μl of ready-to-use tetramethylbenzidine substrate to each well. The plate was then covered and incubated at room temperature for 0.5 hours. One hundred microlitres of stop solution was added to each well. Absorbance was measured at 405 nm using a microtiter plate spectrophotometer followed by an endpoint measurement within one hour.

### Human leukocyte antigen-DR quantification on circulating monocytes

Flow cytometric human leukocyte antigen-DR (HLA-DR) quantification was performed according to the method of Docke and colleagues [21]. In brief, this new method quantifies the number of molecules per monocyte and allows direct comparisons between laboratories.

### Whole blood cell counts

Leukocyte and monocyte cell counts in whole blood were quantified routinely by standardised clinical biochemical methods.

### Statistical analysis

Significance levels between groups were examined using the Kruskal-Wallis test with the Dunn multiple comparison test and Mann-Whitney *U *test where indicated. A *p *value of less than 0.05 was regarded as statistically significant. The time course of the Sepsis-related Organ Failure Assessment (SOFA) scores was analysed by two-way analysis of variance (ANOVA) with repeated measures and Bonferroni *post hoc *analysis. Two-way ANOVA with repeated measures was also used for the time course of hBD2 plasma levels. In contrast, the non-gaussian distribution of *ex vivo *inducible defensin mRNA expression was analysed by the Kruskal-Wallis test. Correlation of the scores with hBD2 inducibility was tested using the Spearman test. Statistical power calculations were performed using an open-access statistical web page [22].

## Results

Sixteen patients with severe sepsis were included in this study. Eight of these patients died from sepsis-induced organ failure. In addition, nine critically ill but non-septic ICU patients and nine healthy volunteers were included. Table 1 shows demographic and clinical data of the patients. Acute Physiology and Chronic Health Evaluation II (APACHE II) and Simplified Acute Physiology Score II scores differed between critically ill non-septic patients and patients with severe sepsis (*p *< 0.05, Mann-Whitney test), whereas age did not (*p *> 0.05, Mann-Whitney test). Underlying diseases for severe sepsis were necrotising fasciitis (*n *= 2; at inclusion, both showed clinical signs of additional pulmonary infection), faecal peritonitis (*n *= 8), and pneumonia (*n *= 6). Finally, all patients with severe sepsis suffered from abdominal or pulmonary infection.

**Table 1:** Demographic and clinical data of critically ill non-septic patients and patients with severe sepsis

	Critically ill non-septic (*n *= 9)	Severe sepsis (*n *= 16)	*p*
Median age (years)	68	55	> 0.05
Median APACHE II score at inclusion	12	29	< 0.05
Median SAPS II score at inclusion	27	60	< 0.05
Mechanically ventilated at inclusion (*n*)	5	16	
Vasopressor treatment at inclusion (*n*)	0	13	
Median IL-6 plasma levels (ng/l)	18	72	< 0.05
Median procalcitonin plasma levels (μg/l)	0.19	2.01	< 0.05
Antibiotic treatment at inclusion (*n*)	7	16	

Statistical differences were calculated by Mann-Whitney test. APACHE II, Acute Physiology and Chronic Health Evaluation II; IL-6, interleukin-6; SAPS II, Simplified Acute Physiology Score II.

Eight of the nine critically ill non-septic patients were in the perioperative period after trauma, abdominal or pharyngeal cancer, or aortic aneurysm rupture with a prolongated postoperative recovery. All of these patients except one were treated with perioperative antibiotic prophylaxis. One patient from this control group suffered from abacterial pancreatitis without antibiotic therapy. None of these patients showed clinically or laboratory signs of infection.

None of the critically ill patients was treated with hydrocortisone. In contrast, 11 patients with severe sepsis were medicated with low-dose hydrocortisone (3 mg/kg body weight per day) at at least one measuring point. All patients with sepsis were treated according to guidelines issued by the Surviving Sepsis Campaign [23].

SOFA score was determined at every time point of blood drawing in the included patients, and APACHE II score was calculated at inclusion. The score differences between the patient groups are illustrated in Table 1. Neither the hBD2 inducibility nor the protein levels showed correlations with APACHE II or SOFA scores (*p *> 0.05, Spearman test; data not shown). hBD2 plasma levels did not show a correlation with the Horowitz quotient, thrombocyte count, creatinin levels, or the need of use of vasopressors (*p *> 0.05, Spearman test; data not shown).

SOFA scores in survivors of severe sepsis were decreased at day five and the last sampling day compared to non-survivors (*p *< 0.05, two-way ANOVA with repeated measures and Bonferroni *post hoc *analysis; data not shown).

**Figure 1:**
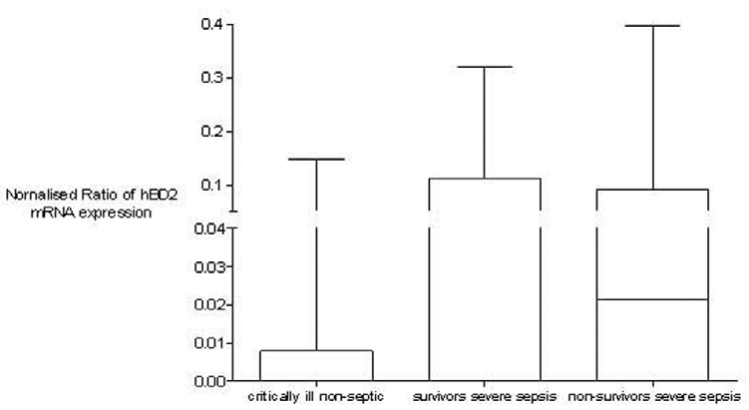
Basic human β-defensin 2 (hBD2) mRNA expression normalised to leukocyte count in critically ill non-septic patients and survivors and non-survivors of severe sepsis shows no differences. No basic mRNA expression was detected in healthy controls (*p *< 0.05, Kruskal-Wallis test with the Dunn multiple comparison test). Data are presented as box-and-whisker plots.

Basic hBD2 mRNA expression was not detectable in peripheral blood cells from healthy controls. The basic hBD2 mRNA expression in survivors and non-survivors of severe sepsis and critically ill patients was normalised to the leukocyte count of every blood sample and showed no differences (*p *> 0.05, Kruskal-Wallis test with the Dunn multiple comparison test; Figure 1).

**Figure 2:**
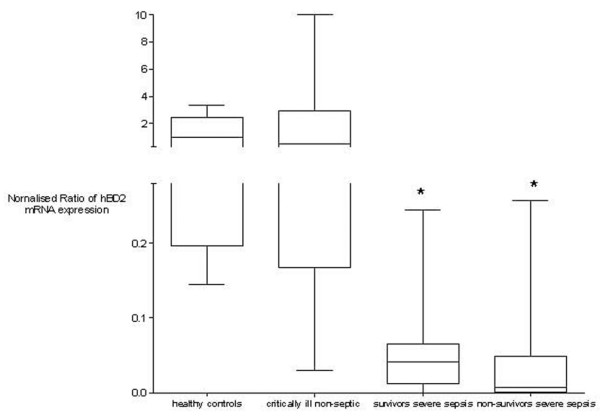
*Ex vivo *human β-defensin 2 (hBD2) inducibility in healthy controls, critically ill non-septic patients, and survivors and non-survivors of severe sepsis. Inducible hBD2 mRNA expression normalised to leukocyte count is decreased in survivors and non-survivors of severe sepsis compared to healthy controls and critically ill non-septic patients (*p < 0.05, Kruskal-Wallis test with the Dunn multiple comparison test). Data are presented as box-and-whisker plots.

**Figure 3:**
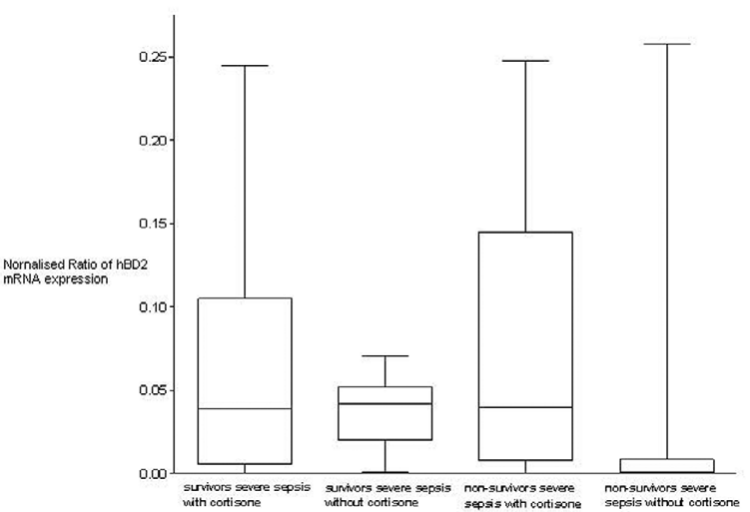
*Ex vivo *human β-defensin 2 (hBD2) inducibility in patients with severe sepsis. Inducible hBD2 mRNA expression normalised to leukocyte count shows no differences in cortisone-treated or non-cortisone-treated patients (*p *> 0.05, Kruskal-Wallis test with the Dunn multiple comparison test). Data are presented as box-and-whisker plots.

In contrast, hBD2 mRNA was detectable in *ex vivo *stimulated cultured whole blood. Endotoxin stimulation (4 hours, 0.5 ng/ml) induced *hBD2 *mRNA expression in all groups and led to low inducibility in patients with severe sepsis. Figure 2 indicates the inducible mRNA expression normalised to leukocyte count at all measured time points. The inducibility in patients with severe sepsis was significantly decreased compared to both other groups (*p *< 0.05, Kruskal-Wallis test with the Dunn multiple comparison test) without differences between survivors and non-survivors of severe sepsis. Despite the limited number of patients, the statistical power of the comparison of hBD2 mRNA inducibility between patients with severe sepsis and controls was 0.95. Hydrocortisone treatment did not impair the leukocyte count-normalised hBD2 mRNA inducibility in patients with severe sepsis (*p *> 0.05, Kruskal-Wallis test with the Dunn multiple comparison test; Figure 3).

**Figure 4:**
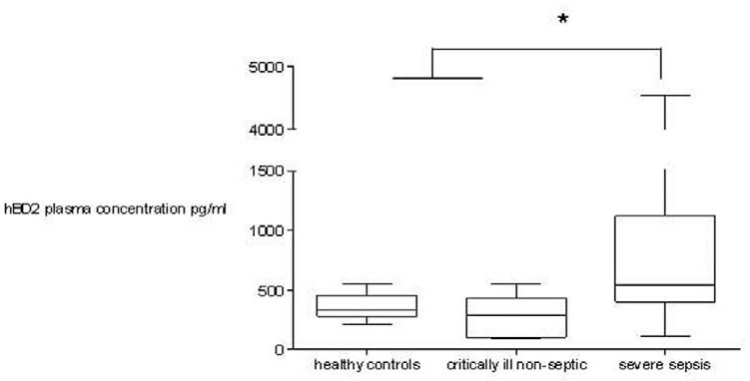
Human β-defensin 2 (hBD2) plasma protein concentration in healthy controls, critically ill non-septic patients, and patients with severe sepsis. Plasma concentration in healthy controls and critically ill non-septic patients was significant lower compared to patients with severe sepsis (*p < 0.05, Kruskal-Wallis test with the Dunn multiple comparison test). Data are presented as box-and-whisker plots.

In addition, hBD2 protein concentration was quantified in plasma at all included time points. hBD2 plasma concentrations in non-septic critically ill patients and healthy controls were significantly lower compared to patients with severe sepsis (*p *< 0.05, Kruskal-Wallis test with the Dunn multiple comparison test; Figure 4). The comparison of hBD2 plasma levels reached statistical significance at a power of 0.98. No differences were detected between survivors and non-survivors of severe sepsis.

**Figure 5:**
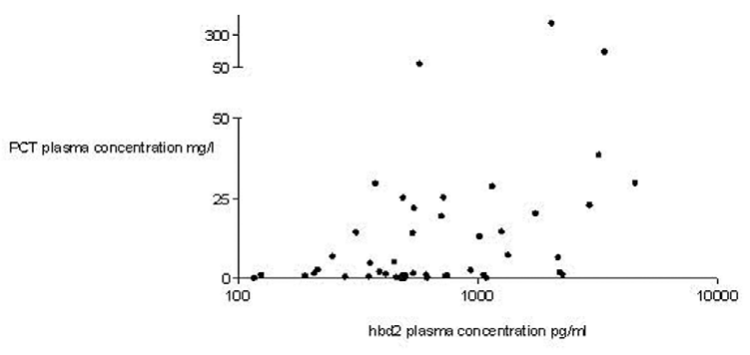
Human β-defensin 2 (hBD2) plasma protein and procalcitonin (PCT) levels showed a significant correlation in patients with severe sepsis (*p *< 0.005, Spearman test).

hBD2 protein levels showed no correlation with interleukin (IL)-6 plasma levels in septic patients (*p *> 0.05, correlation coefficient *r *= -0.041, Spearman test; data not shown). In contrast, procalcitonin (PCT) plasma levels and hBD2 protein plasma levels showed a positive correlation in patients with severe sepsis (*p *< 0.005, correlation coefficient *r *= 0.4203, Spearman test; Figure 5).

**Figure 6:**
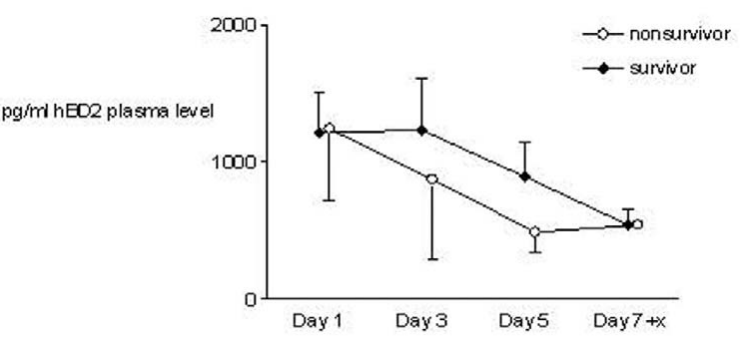
Human β-defensin 2 (hBD2) plasma protein concentration at different time points in patients with severe sepsis. Time course of hBD2 plasma protein concentration in survivors and non-survivors of severe sepsis showed no statistical differences (*p *> 0.05, two-way analysis of variance with repeated measures). Data are presented as mean ± standard error of the mean.

The time course of hBD2 plasma protein concentration in patients with severe sepsis did not differ significantly between survivors and non-survivors, however it showed considerable variation between survivors and non-survivors (*p *> 0.05, two-way ANOVA with repeated measures; Figure 6).

HLA-DR quantification was performed in patients with severe sepsis and non-septic critically ill patients. HLA-DR molecules on circulating monocytes per cell in non-septic critically ill patients were significantly higher compared to patients with severe sepsis (*p *< 0.05, Mann-Whitney *U *test; data not shown).

## Discussion

The present investigation shows the novel finding of impaired *hBD2 *gene inducibility in peripheral cells and elevated plasma protein concentration in patients with severe sepsis compared to non-septic critically ill patients and healthy controls. The meaning of β-defensins for the defence of infections is based on well-described antimicrobial activities [24,25]. In addition, β-defensins induce prostaglandin D_2 _production, degranulate mast cells, and present chemotactic activities on CCR6-positive dendritic cells [6,26]. In mice, additional immunomodulatory effects have been reported [6,27,28]. These data indicate their involvement in innate immunity. These reported effects suggest regulatory or mediatory defensin functions. The role of antibiotic peptides in the pathogenesis of Crohn's colitis, cystic fibrosis, and panbronchiolitis has been described clearly. An effective defence related to levels and inducibility of defensins has been reported [17,29-33].

The elevated plasma levels of hBD2 in patients with severe sepsis indicate a higher activity of inflammation compared to non-septic individuals. Proinflammatory cytokines such as IL-1 and tumour necrosis factor induce hBD2 gene expression in alveolar macrophages and monocyte-derived epidermis cells (IL-1) [10,12]. These proinflammatory cytokines, which are frequently elevated in severe sepsis, are potentially involved in the upregulation of systemic hBD2 release in sepsis as well. The decreased hBD2 inducibility in peripheral blood cells was not associated with decreased plasma levels, suggesting that peripheral blood cells do not represent the exclusive source of released hBD2 protein *in vivo*.

The hBD2 plasma concentration in healthy controls agrees with findings from other investigations [17,34]. It should be taken into account that circulating endothelial cells or reticuloendothelial cells also represent a possible source of hBD2 [35]. The results for hBD2 mRNA inducibility and the basic protein plasma levels showed no significant differences between healthy controls and critically ill non-septic patients. Median PCT levels were in normal range, indicating a lack of systemic infection, whereas a median IL-6 of 18 ng/l (normal is below 15 ng/l) suggested minor systemic inflammatory activation in the non-septic critically ill patient group. For gene activation of hBD2 and IL-6, the transcription factor nuclear factor-kappa B (NF-κB) is crucial. The low IL-6 levels in the critically ill non-septic group provide a hint for, but are not proof of, low NF-κB activation in this group. This minor activation showed no influence on hBD2 inducibility or protein levels compared to healthy controls. Only the systemic infection in the severe septic patient group led to changes in gene inducibility and plasma levels. These results underline a specific impact of systemic infections on *hBD2 *gene expression and plasma levels.

The decreased hBD2 mRNA inducibility in peripheral blood cells of patients with severe sepsis could mirror a serious inhibition of innate immune function. But given that the detected plasma concentrations were lower than required for bactericidal/antiviral activity, antimicrobial peptides may not exert their antimicrobial effects via the bloodstream [36-38]. However, innate immunity may be impaired not only due to the lack of direct antimicrobial activity but because of limited immunomodulating effects of defensins.

This immunological imbalance occurring in severe sepsis can be monitored, among other ways, by HLA-DR quantification on circulating monocytes. In this manner, the immune competence of monocytes can be assessed. It is well established that monocytes with diminished HLA-DR expression are inhibited in some of their main tasks (for example, antigen presentation and mediator production) [39,40]. Indeed, the investigated patients with severe sepsis showed signs of immunodepression by decreased HLA-DR expression on circulating monocytes. This finding underlines that sepsis may contribute to the impaired hBD2 inducibility as reported in the present investigation.

In this investigation, hydrocortisone treatment did not impair hBD2 inducibility in patients with severe sepsis. However, at the present time, there are no consistent data on the influence of steroid medication on hBD2 inducibility [41-43].

An individual's age can modulate immune function. Activities of cellular components of innate immunity are impaired at different levels [44-46]. To date, no data assessing antimicrobial peptide gene expression in the elderly have been collected. However, in insects, antimicrobial peptide gene expression increases with age [47]. The median age of the control group was significantly lower compared to both other groups. There was no significant difference of the median age between the critically ill and the septic patients. Therefore, the differences between the critically ill and the septic patients concerning hBD2 mRNA inducibility and plasma levels cannot be explained by differences in age.

## Conclusion

hBD2 inducibility in leukocytes from patients with severe sepsis is decreased. This special part of innate immunity is influenced by severe sepsis. The downregulation of inducibility may contribute to the complex immunological imbalance occurring in patients with severe sepsis.

The importance of plasmatic hBD2 for patients with severe sepsis is unclear. In particular, knowledge of the interaction with mediators and effectors of the immune system is scarce but of prime importance. To date, the antimicrobial and immunomodulatory activities of hBD2 have been tested only in *ex vivo *settings with limited numbers of additional co-factors. However, *in vivo*, hBD2 is an integral component of a set of effectors that function together in the innate immune line of defence.

## Key messages

• *Ex vivo *endotoxin hBD2 inducibility in leukocytes was decreased in patients with severe sepsis compared to healthy controls and critically ill non-septic patients.

• hBD2 plasma levels were elevated in the severe sepsis group compared to both other groups.

• hBD2 inducibility and plasma levels showed no differences between survivors and non-survivors of severe sepsis.

## Abbreviations

AMV = avian myeloblastosis virus; ANOVA = analysis of variance; APACHE II = Acute Physiology and Chronic Health Evaluation II; BSA = bovine serum albumin; C_p _= crossing point; hBD2 = human β-defensin 2; hHPRT = human hypoxanthine phosphoribosyl-transferase; HLA-DR = human leukocyte antigen-DR; ICU = intensive care unit; IL = interleukin; NF-κB = nuclear factor-kappa B; PCR = polymerase chain reaction; PCT = procalcitonin; SOFA = Sepsis-related Organ Failure Assessment.

## Competing interests

The authors declare that they have no competing interests.

## Authors' contributions

MB participated in the coordination and design of the study and performed the statistical analysis. QC participated in the design of the study and worked on *ex vivo *gene inducibility and protein quantification. LEL participated in the statistical analysis, planning of the study, and selection of patients and helped to draft the manuscript. SK participated in the coordination of the study and the protein quantification. SW participated in *ex vivo *stimulations and the design of the study. J-CS participated in the coordination of the study and in generating the manuscript. ML participated in the design and coordination of the study. AH participated in revising the manuscript and in the design of the study. FS initiated the study and gave major advice for the design of the study and the methods used. All authors read and approved the final manuscript.
